# COVID-19: An Emerging Culprit of Inflammatory Arthritis

**DOI:** 10.1155/2021/6610340

**Published:** 2021-04-26

**Authors:** Muhammad Shariq Mukarram, Muhammad Ishaq Ghauri, Sehrish Sethar, Nasir Afsar, Amir Riaz, Khizra Ishaq

**Affiliations:** ^1^Department of Internal Medicine, Jinnah Medical College Hospital, Street 6, Sector 7-A, Korangi Industrial Area, Karachi, Pakistan; ^2^Department of Medicine, Liaqat National Hospital, Karachi, Pakistan

## Abstract

Arthralgia is one of the most common symptoms that occur in patients with COVID-19. About 15% of patients present with arthralgia at some point. Although COVID-19 seems to attack the musculoskeletal system (muscles and joints) in its infective and postinfective stage causing inflammatory arthritis, not much is known about the rheumatic manifestations of this infection. In this case series of 5 patients, we discuss the occurrence of bilaterally symmetrical polyarthritis in patients, previously free from any rheumatic disease, after encountering COVID-19 infection. The musculoskeletal manifestations in these patients phenotypically resembled rheumatoid arthritis. These patients were treated successfully with low-dose glucocorticoids and disease-modifying antirheumatic drugs (DMARDs).

## 1. Introduction

Coronavirus disease 2019 (COVID-19) is an emerging pandemic that is caused by severe acute respiratory distress coronavirus 2 (SARS-CoV-2). The virus mainly affects the respiratory system but has indirect effects on multiple organ systems including the musculoskeletal system [1]. Fatigue, arthralgia, and myalgia have been identified as common COVID-19 symptoms with considerable musculoskeletal dysfunction in some patients, but long-term follow-up and their prevalence have yet not been investigated [1–3].

A very recent study collected data of the past 5 months, from different studies published worldwide, to detect the frequency of musculoskeletal symptoms in COVID-19 patients. It included a total of 54 studies (12,045 patients), mostly from China, and found that, amongst the musculoskeletal symptoms, fatigue and arthralgia/myalgia were the most common symptoms [4]. Eight studies reported having a prevalence of greater than 50% of patients with fatigue [5–12] while three reported higher values of arthralgia/myalgia [13–15]. A retrospective single-center analysis conducted in Wuhan, China, included 84 confirmed cases of SARS-CoV-2 infection. Nearly two-thirds of patients had myalgia or fatigue [5]. Another city in China, Chengdu, evaluated the epidemiological characteristics of 99 test-positive cases who were admitted to the hospital. It was seen that after cough, fatigue (73%) was the most common symptom in these patients [10]. An article published in the New England Journal of Medicine discussed the clinical characteristics of COVID-19 in New York City. According to it, myalgias (23.8%) were not uncommon among 393 patients [11].

The use of nonsteroidal anti-inflammatory drugs (NSAIDS) is very common in COVID-19. Therefore, the persistence of musculoskeletal complaints is even more worrying because it may indicate that inflammatory reactions can overcome the anti-inflammatory effects of these drugs. We hereby present a case series of 5 patients who presented with inflammatory arthritis (symmetrical/polyarticular) as a sequela of COVID-19 infection. The current literature on inflammatory manifestations, especially symmetrical polyarthritis, is scarce.

These patients presented to the rheumatology outpatient clinic complaining of new-onset joint pain after recovering from SARS-CoV-2 infection. All patients were suffering from polyarthritis showing symmetrical distribution, including large and small joints. The patient characteristics and findings are summarized in Table 1. The musculoskeletal ultrasound scan showed evidence of symmetrical synovitis. To the best of our knowledge, this is the first case series of patients who recovered from COVID-19 developing bilaterally symmetrical polyarthritis which phenotypically resembled rheumatoid arthritis.

## 2. Case Presentation

### 2.1. Case 1

A 65-year-old female, known case of hypertension, who had been diagnosed and treated as COVID-19 infection two months ago presented to the rheumatology outpatient clinic with complaints of pain in both hands, including the small joints of hands, for the last one month. The pain was intermittent and was more marked in the morning upon waking up. She used nonsteroidal anti-inflammatory drugs (NSAIDs) for this which provided her temporary relief.

**Figure 1:**
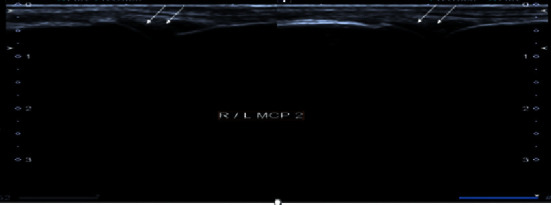
Grayscale images of right and left 2^nd^ metacarpophalangeal joint demonstrating moderate synovial thickening (white arrows). The findings are consistent with Grade 2 synovitis.

**Figure 2:**
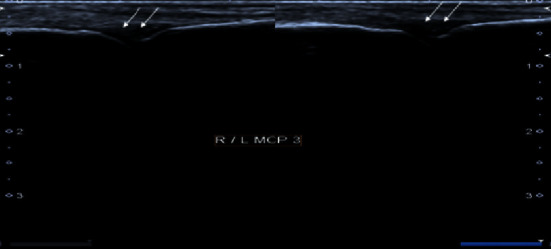
Grayscale images of bilateral 3^rd^ metacarpophalangeal joint demonstrating moderate synovial thickening (white arrows). The findings are consistent with Grade 2 synovitis.

On examination, there was tenderness present bilaterally in her proximal interphalangeal (PIP) and wrist joints. Upon investigations, her RA factor and anti-CCP were negative. Before this, she has never had an episode of similar joint pain. She was advised musculoskeletal ultrasound, findings of which are as follows (Figures 1 and 2 ):

- Grade 2 synovitis in bilateral metacarpophalangeal (MCP) joints 2 and 5 and Grade 1 synovitis in bilateral PIP joints 2 to 4
- Grade 2 synovitis in both wrists
- Power Doppler negative
- No bony erosions seen

### 2.2. Case 2

A 35-year-old male, healthcare worker with no prior known comorbid, presented to the rheumatology clinic with complaints of pain in multiple joints for the last 2 weeks. Six weeks earlier, the patient was diagnosed with COVID-19 infection after he had contracted exposure with an affected individual.

**Figure 3:**
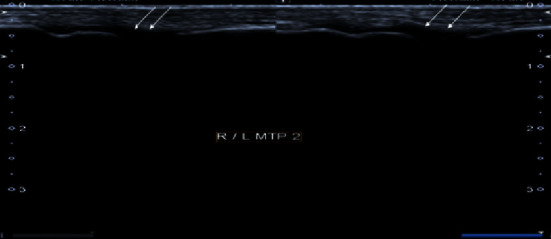
Grayscale images of bilateral 2^nd^ metatarsophalangeal joint demonstrating moderate synovial thickening (white arrows). The findings are consistent with Grade 2 synovitis.

**Figure 4:**
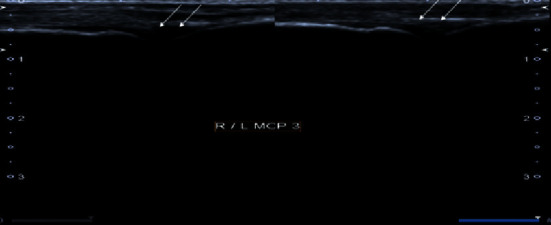
Grayscale images of bilateral 3^rd^ metacarpophalangeal joint demonstrating moderate synovial thickening (white arrows). The findings are consistent with Grade 2 synovitis.

On examination, he had pain and tenderness in his wrist and MCP joints bilaterally. There was evidence of active inflammation (warmth, tenderness, and swelling) in both his ankle joints. His RA factor and anti-CCP turned out to be negative. Results provided by musculoskeletal ultrasound are as follows (Figures 3 and 4 ):

- Grade 2 synovitis in bilateral MCP joints
- Grade 1 synovitis in bilateral PIP joints 2 to 5
- Grade 2 synovitis in both wrists
- Bilateral ankle Grade 2 synovitis
- Grade 2 synovitis in bilateral metatarsophalangeal (MTP) joints 2 and 5
- Power Doppler negative
- No bony erosions seen

### 2.3. Case 3

**Figure 5:**
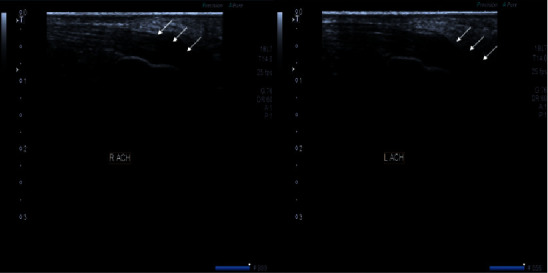
Extensor tendons of bilateral Achilles show thickening and hypoechogenicity representing tendonitis (white arrows).

**Figure 6:**
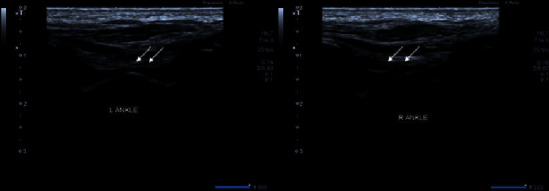
Grayscale images of bilateral ankle joints demonstrating moderate synovial thickening. The findings are consistent with Grade 2 synovitis (white arrows).

**Figure 7:**
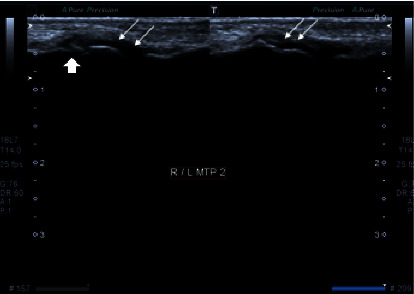
Grayscale images of bilateral 2^nd^ metatarsophalangeal joint demonstrating moderate synovial thickening (white arrows). The findings are consistent with Grade 2 synovitis. There is subtle irregularity seen in the bony cortex of the underlying bone on the right side suggesting erosions (block arrow).

A 25-year-old female otherwise healthy, a doctor by profession, presented to the rheumatology outpatient clinic with complaints of generalized joint pains for the last 15 days, associated with morning stiffness of 40 minutes. According to the patient, she was diagnosed with COVID-19 infection 2 months back after a history of recent travel. Her pain temporarily subsided on potent analgesics. On examination, there were signs of inflammation (swelling, warmth, and tenderness) over her ankles and MTP joints bilaterally. There was also tenderness noted in her MCP and wrist joints bilaterally. Her RA factor and anti-CCP were negative. Musculoskeletal ultrasound findings are as follows (Figures 5–7 ):

- Grade 1 synovitis in bilateral MCP joints 2 to 5 and Grade 1 synovitis in bilateral PIP joints 2 to 5
- Grade 1 synovitis in both wrists
- Bilateral ankle Grade 2 synovitis and bilateral Achilles tendonitis
- Grade 2 synovitis in bilateral MTP joints 2 and 5
- Power Doppler negative
- No bony erosions seen

### 2.4. Case 4

A 32-year-old female with no known comorbid had suffered from COVID-19 infection 2.5 months ago. She presented to the rheumatology clinic with complaints of pain in small joints of her hands for the last 6 weeks which started 2 weeks after completely recovering from her disease process. Initially, the pain was intermittent but later became continuous, associated with morning stiffness of 20 minutes, due to which she was compelled to seek medical attention.

**Figure 8:**
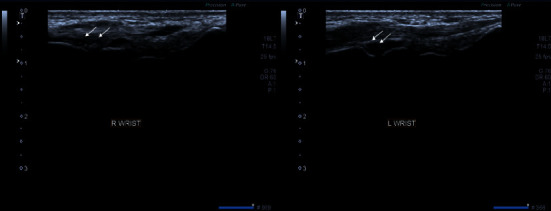
Grayscale images of both wrist joints demonstrating moderate synovial thickening (white arrows). The findings are consistent with Grade 2 synovitis. Extensor tendons of both wrist joints also show thickening and hypoechogenicity representing tendonitis.

**Figure 9:**
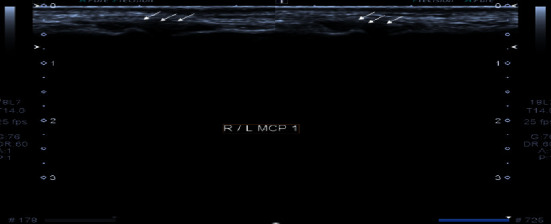
Grayscale images of bilateral 1^st^ metacarpophalangeal joint demonstrating mild synovial thickening (white arrows). The findings are consistent with Grade 1 synovitis.

On examination, there was tenderness and swelling in her PIP and wrist joints bilaterally. Her RA factor and anti-CCP were negative. Musculoskeletal ultrasound findings are as follows (Figures 8 and 9 ):

- Grade 2 synovitis in bilateral MCP joint 2 and Grade 2 synovitis in bilateral PIP joints 2 to 5
- Grade 2 synovitis in both wrists
- Power Doppler negative
- No bony erosions seen

### 2.5. Case 5

A 40-year-old male patient who is a known case of type II diabetes mellitus on oral hypoglycemic drugs had presented to the rheumatology outpatient department after recovering from COVID-19 infection which he had encountered 1.5 months ago. His chief complaint was a pain in both his wrist joints for the past 2 weeks, more severe in the morning.

**Figure 10:**
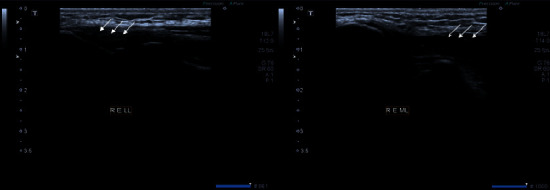
Tendon of right elbow joint shows thickening and hypoechogenicity representing tendonitis (white arrows).

**Figure 11:**
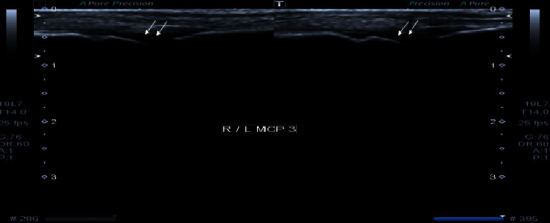
Grayscale images of 3^rd^ metacarpophalangeal joint demonstrating moderate synovial thickening. The findings are consistent with Grade 2 synovitis (white arrows).

On examination, there was tenderness in a few of the MCP joints bilaterally, but the wrist joint in both hands was markedly tender and swollen. RA factor and anti-CCP turned out to be negative. Musculoskeletal ultrasound was consistent with the following findings (Figures 10 and 11 ):

- Grade 2 synovitis in bilateral MCP joints 1–5
- Grade 2 synovitis in both wrists
- No synovitis in any of the PIP joints
- Power Doppler negative
- No bony erosions seen

## 3. Clinical Course and Therapeutic Intervention

All of these 5 patients had remained clinically stable during their course of the primary disease with no event/episode of hypoxia (SPO_2_ > 95%). They were advised to self-quarantine and were treated by their primary healthcare physician.

Based on clinical examination and finding of the musculoskeletal ultrasound, these patients were diagnosed as seronegative post-COVID-19 inflammatory arthritis (polyarticular) which phenotypically resembled rheumatoid arthritis. Interestingly, the specific pattern observed in these patients was the involvement (synovitis) of small joints of hands and wrist joints bilaterally which was common in all five patients. This very much resembles the clinical presentation of rheumatoid arthritis. All patients were prescribed a common treatment plan. They were commenced on low-dose tapering corticosteroid (prednisone 10 mg/d) along with etoricoxib. Amongst disease-modifying antirheumatic drugs (DMARDs), leflunomide (20 mg/d) and hydroxychloroquine (400 mg/d in 2 divided doses) were commenced. Patients were advised to follow up after three weeks.

## 4. Discussion

The clinical features and mechanisms of COVID-19 musculoskeletal manifestations require deep analysis. These symptoms are thought to mainly arise from inflammatory and/or immune responses. This hypothesis is based on the involvement of proinflammatory markers (IL-6 and TNF-*α*) released in alveolar and musculoskeletal inflammation [4, 16].

Inflammatory reactive arthritis (ReA) involving different joints has well been reported from different parts of the world as a complication occurring after COVID-19 infection. The first case of ReA was reported in a 73-year-old patient who was diagnosed and treated for SARS-CoV-2 infection. Eight days later, he developed signs of inflammation in his left first metatarsophalangeal, proximal, and distal interphalangeal joints. After two days, similar findings appeared in the right second proximal and distal interphalangeal joints. The screening laboratory tests for arthritis were negative, similar to all our patients. Because of typical findings at case presentation, the patient was diagnosed with reactive arthritis caused by COVID-19 [17].

Another case is of a 50-year-old male who was admitted with COVID pneumonia. His disease progressed to ARDS and was intubated. On day 11, he was extubated, subsequently completing a 14-day course of favipiravir. After being started on physical therapy on day 21, he developed acute bilateral arthritis in his ankles with mild enthesitis in his right Achilles tendon in the absence of any kind of rash, conjunctivitis, urethritis, or any preceding diarrhea. The aspiration of the synovial fluid revealed inflammatory fluid with a negative culture. The patient was diagnosed and treated for ReA [18].

The Lancet Rheumatology published a case of a 58-year-old white woman from Europe who had COVID-19 of mild intensity. A couple of weeks later, she reported inflammation of her ankle joint with a small rise in the inflammatory markers. An ultrasound examination showed a thickening of the synovial membrane of the ankle with inflammation of the Achilles tendon [19]. All five patients in our case series were diagnosed by the same choice of investigation (musculoskeletal ultrasound).

The knee joint is rarely involved but has the potential of being affected as a part of reactive arthritis. Ghauri et al. lately reported a case of a 34-year-old male who developed reactive arthritis (right knee) ten days after being diagnosed with COVID-19. Due to severe pain and inflammation, he was injected with intra-articular glucocorticoid [20].

Chikungunya virus is also commonly associated with postviral arthralgia. A similar phenomenon of postviral inflammatory arthritis was reported by Ghauri et al. after a Chikungunya virus epidemic broke out in Pakistan in 2017 [21]. A large cross-sectional study was carried out by Dr. Chang on patients with chikungunya who developed inflammatory arthritis, but the synovial fluid did not reveal the presence of the virus. Based on this, he explains arthritis as being secondary to the induction of host autoimmunity justifying the role of immunomodulating drugs in its treatment [22].

Very interestingly, the development of rheumatoid arthritis in relation to coronavirus has been studied. The authors of a Korean study observed that infections with endemic human coronavirus, parainfluenza virus, and metapneumovirus coincided with an interesting rate of development of rheumatoid arthritis [23]. To date, there is a lack of sufficient data to indicate that people develop autoimmune inflammatory arthritis after being infected with SARS-CoV-2 infection.

The musculoskeletal ultrasound (MSUS) has been a revolutionary tool in the field of rheumatology and is seven times more sensitive than plain radiography, allowing earlier diagnosis and intervention of progressive diseases. The best advantage is the rapid method and can be performed in a clinic with the assessment of multiple joints at a time. It is a noninvasive radiation-free technique with less scan time [24]. The presence of synovitis in our cases was confirmed by MSUS.

This is the first case series of patients presenting with polyarthritis, clinically and radiologically mimicking seronegative inflammatory arthritis, with a recent history of COVID-19 infection.

## 5. Conclusion

To date, there is not sufficient data to indicate that people develop autoimmune inflammatory arthritis after being affected by SARS-CoV-2. Therefore, our case series adds a substantial amount of evidence to this hypothesis.

## Figures and Tables

**Table 1:** Patient characteristics and findings.

Parameters	Case 1	Case 2	Case 3	Case 4	Case 5
Age	65	35	25	32	40

Sex	Female	Male	Female	Female	Male

Comorbidity	Hypertension	None	None	None	Type 2 diabetes mellitus

History of COVID-19 infection (weeks) prior to presentation	8	6	8	10	6

Distribution of joint involvement	Both hands, including small joints	Both hands, including small joints	Generalized	Both hands, including small joints	Both hands, including small joints

Duration of joint pain	4 weeks	2 weeks	15 days	6 weeks	2 weeks

Physical examination	Bilateral tenderness in wrists and PIP joints	Bilateral tenderness in wrists and MCP joints	Bilateral tenderness in wrists, MCP, ankles, and MTP joints	Bilateral tenderness in wrists and PIP joints	Bilateral tenderness in wrists and few MCP joints

Synovitis in the musculoskeletal ultrasound scan	Grade 2 synovitis in bilateral wrists and MCP joints 2 and 5 Grade 1 synovitis in bilateral PIP joints 2–4	Grade 2 synovitis in bilateral wrists, MCP joints 2–5, ankles, and MTP joints 2 and 5 Grade 1 bilateral PIP joints 2–5	Grade 2 synovitis in bilateral ankles and MTP joints 2 and 5 Grade 1 synovitis in bilateral wrist, PIP joints 2–5, and MCP joints 2–5	Grade 2 bilateral synovitis in wrists and MCP joints 2, and PIP joints 2–5	Grade 2 synovitis in bilateral wrists and MCP joints 1–5

PIP: proximal interphalangeal; MCP: metacarpophalangeal; MTP: metatarsophalangeal.

## Data Availability

No data were used to support the findings of this study.
